# Characteristics of the Gut Microbiome and IL-13/TGF-β1 Mediated Fibrosis in Post-Kasai Cholangitis of Biliary Atresia

**DOI:** 10.3389/fped.2021.751204

**Published:** 2021-11-08

**Authors:** Lingdu Meng, Jia Liu, Junfeng Wang, Min Du, Shouhua Zhang, Yanlei Huang, Zhen Shen, Rui Dong, Gong Chen, Shan Zheng

**Affiliations:** ^1^Department of Pediatric Surgery, Children's Hospital of Fudan University, Shanghai Key Laboratory of Birth Defect, Key Laboratory of Neonatal Disease, Ministry of Health, Shanghai, China; ^2^Department of General Surgery, Jiangxi Provincial Children's Hospital, Nanchang, China

**Keywords:** biliary atresia, cholangitis, gut microbiome, Kasai procedure, *Klebsiella pneumoniae*, organoid

## Abstract

**Aims:** Cholangitis in biliary atresia (BA), which accelerates liver fibrosis progression, is among the most common serious complications after Kasai surgery; however, its etiology remains elusive. Gut microbiome migration may contribute to post-Kasai cholangitis. Further, there is no appropriate model of BA post-Kasai cholangitis for use in investigation of its pathogenesis.

**Methods:** We explored the characteristics of gut microbiome in patients with BA before and after Kasai procedure based on 16S rDNA sequencing. We isolated the dominant strain from patient stool samples and established an *in vitro* model by infecting patient-derived liver organoids. Bulk RNA-seq was performed, and we conducted qPCR, ELISA, and western blot to explore the mechanism of fibrosis.

**Results:** Gut microbiome diversity was lower in patients after, relative to before, Kasai procedure, while the relative abundance of *Klebsiella* was higher. Patients who developed cholangitis within 1 month after discharge tended to have simpler gut microbiome composition, dominated by *Klebsiella*. *Klebsiella pneumoniae* (KPN) was isolated and used for modeling. RNA-seq showed that BA liver organoids expressed markers of hepatic progenitor cells (*KRT19, KRT7, EPCAM*, etc.) and that organoids were more stable and less heterogeneous among individuals than liver tissues. After infection with KPN, gene expression patterns in BA liver organoids were enriched in pathways related to infection, apoptosis, and fibrosis. Preliminary experiments indicated the presence of IL-13/TGF-β1-mediated fibrosis in post-Kasai cholangitis.

**Conclusions:** Our findings using a newly-developed model, demonstrate a key role for *Klebsiella*, and a potential mechanism underlying fibrosis in post-Kasai cholangitis, mediated by the IL-13/TGF-β1 pathway.

## Introduction

Biliary atresia (BA) is a common hepatobiliary disease in infancy, which is characterized by progressive intrahepatic and/or extrahepatic bile duct obstruction and liver fibrosis. Without effective intervention, children with BA do not often survive beyond 2 years of age (1). The Kasai procedure is currently the standard treatment for BA, and cholangitis is among the most common serious complications following this approach, with an incidence of approximately 30–70%, mostly within 6 months after surgery. Post-operative fever of unknown cause, body temperature >38°C, lightened stool color, increased bilirubin, and increased C-reactive protein (2, 3) are often symptoms of cholangitis. Recurrent cholangitis affects bile drainage, potentially leading to cholestasis, bile duct sclerosis, and aggravation of liver fibrosis, which are important in the prognosis of patients with BA (4). More frequent cholangitis occurrence results in more severe liver fibrosis and worse prognosis. Further, early-onset cholangitis [within 1 month after surgery (5, 6)] increases the risk of recurrent cholangitis (7).

The etiology and pathogenesis of post-Kasai cholangitis remain unclear; however, it is widely believed to be related to migration of the gut microbiome. Pathogenic bacteria associated with post-Kasai cholangitis are mostly from the intestinal microbial community, among which *Escherichia coli* and *Pseudomonas aeruginosa* are the most common (7, 8). In recent years, with the continuous development of high-throughput sequencing technology, changes in the gut microbiome during the pathogenesis of various liver diseases have been revealed (9, 10). In primary sclerosing cholangitis, non-alcoholic cirrhosis, and other fibrosis-related liver diseases, there are changes in the diversity and structure of the intestinal flora, and some characteristic microbiome features (11, 12). In addition, the intestinal flora is crucial to bile metabolism, which has a specific impact on cholestatic and metabolic liver diseases (13, 14). Hence, the intestinal flora may be related to the occurrence and development of various liver diseases through regulating immunity and metabolism. As migration of intestinal microorganisms may be the factor that initiates the occurrence and development of cholangitis after Kasai procedure, changes in the intestinal flora may be important in the development of liver inflammation and fibrosis.

It has been difficult to establish an effective cell or animal model for post-Kasai cholangitis. In recent years, organoids have been applied to study various tissues (including organs as well as tumors) and to generate many disease models (15, 16). Organoids can not only partially replicate the physiological and biochemical functions of the source organ, but also retain the genetic characteristics of the host (17, 18), making them ideal candidates for establishing an *in vitro* model of post-Kasai cholangitis.

Here, we explored the dynamic changes in the gut microbiome of children with BA and post-Kasai cholangitis using high-throughput sequencing technology and investigated the potential role of the intestinal flora in the development of the disease by using key bacterial strains to treat BA liver organoids and establish a model of post-Kasai cholangitis. Further, we studied the role of the intestinal flora in the onset of cholangitis and liver fibrosis promotion. Our findings provide new insights with potential to inform future research.

## Materials and Methods

### Subject Recruitment

Participants enrolled in this study (*n* = 65) were patients admitted to the Department of Pediatric Surgery of the Children's Hospital of Fudan University from December 2016 to January 2021, and diagnosed with type III BA according to the criteria described in the Supplementary Material. Stool samples (*n* = 39) and liver tissues (*n* = 26) were collected from patients with BA. In addition, five para-hepatoblastoma (unaffected tissue) samples from patients were included as healthy controls.

### Patient Groups

Samples collected for stool DNA extraction (*n* = 39) were divided into two groups: Group_S1 comprised 16 samples collected 1 day before the Kasai procedure and Group_S2 included 23 samples collected 14 days after Kasai procedure, without cholangitis occurrence. Patients in Group_S2 were followed up for 1 month after discharge, and further separated into three subgroups according to the occurrence of post-Kasai cholangitis: Group_S2A (patients did not develop cholangitis), Group_S2B (patients had ≥1 episode of cholangitis), and Group_S2C (data not recorded). All patients received the same antibiotic therapy in the hospital. Information about patient groups is shown in Supplementary Table 1.

### Stool Sample Collection and DNA Extraction

Stool samples were scraped from baby diapers using a sterile swab in the hospital and immediately frozen at −80°C. Genomic DNA was extracted from fecal samples using a QIAamp 96 PowerFecal QIAcube HT kit (51531, QIAGEN, Germany). Quality and quantity of DNA were checked by 1.0% (w/v) agarose gel electrophoresis. All DNA samples were stored at −80°C until further processing.

### 16s rDNA Sequencing and Data Quality Control

Two-step PCR amplification was used for 16S rDNA gene library construction. In the first PCR reaction, the 16S rDNA V3–V4 region was amplified using the primer set, 343F (5′-TACGGRAGGCAGCAG-3′) and 798R (5′-AGGGTATCTAATCCT-3′). In the second PCR reaction, dual indices and Illumina sequencing adapters were ligated to the amplicon. PCR products were pooled, confirmed by 2% agarose gel electrophoresis, cleaned using Agencourt AMPure XP Reagents beads (Bechman Coulter, Brea, CA, USA), according to the manufacturer's protocol, and sequenced using the Illumina MiSeq System (Illumina Inc., USA).

Sequencing primers and low-quality bases were removed from raw reads using the Trimmomatic software package (19). Paired-end reads were overlapped and merged using FLASH (20). Assembly parameters were: 10 bp minimum overlap, 200 bp maximum overlap, and 20% maximum mismatch rate. Clean tags were obtained after further trimming using QIIME (21). Finally, chimeras were removed using the UCHIME software (22), which resulted in high-quality valid tags.

Operational taxonomic units (OTUs) were classified using Vsearch (23) at a 97% similarity threshold. Representative sequences, which were those with the greatest abundance, were annotated and blasted against the SILVA database (v123) using the RPD classifier (24). An OTU table was established for subsequent analysis.

### Microbiome Analyses

Alpha diversity (the richness of a sample in terms of the diversity of OTUs observed in it) was estimated from observed OTUs, as well as the Chao1 richness estimator, and Shannon and Simpson diversity indices. The relative abundance of different genera in each group was compared using the Mann-Whitney test. Beta diversity (distance between samples based on differences in OTUs present in each sample) was measured using principal component analysis, as well as principal coordinates and non-metrical multidimensional scaling analyses, based on Binary Jaccard analysis; statistical significance was assessed using the ADONIS test, a multivariate analysis of variance, based on permutation, or non-parametric MANOVA. Linear discriminant analysis effect size (LEfSe) (25), a method for biomarker discovery, was used to determine the genera that best characterized each study group. Higher LEfSe scores indicate greater consistency in the differences in relative abundance between taxa in the groups analyzed. Random Forest is a machine learning algorithm for classification and can measure the relative importance of each feature on the prediction.

### Isolation, Culture, and Identification of Bacteria

Fresh stool samples were transferred from participant diapers to sterile tubes, using sterile cotton swabs, then stored in a refrigerator at 4°C. Specimens were inoculated and cultured on MacConkey Agar on the same day using the quadrant streaking plate method. Then, plates were incubated aerobically at 35–37°C for 18–24 h. Isolated colonies were identified by mass spectrometry testing, and *Klebsiella* genus strains were selected for subculture. After passaging 2–3 times to obtain a single stable strain, samples were stored in fresh LB medium at −20°C.

### Generation of Organoids From Liver With BA

Wedge liver biopsies were collected from children with type III BA under sterile conditions, stored in Basal Medium [Advanced DMEM/F-12; 1% Penicillin/Streptomycin (P/S); 1% GlutaMAX], and used to generate organoids within 24 h. Liver organoid generation, passage, and freezing followed the protocols described by Laura Broutier et al. (26).

Liver tissues were minced into pieces and washed twice with cold Wash Medium [DMEM (high glucose); 1% FBS; 1% P/S]. When the pieces settled, the supernatant was discarded, pieces transferred into 4–5 ml per g prewarmed liver digest solution (EBSS, Earle's Balanced Salt Solution; 2.5 mg/ml Collagenase D; 0.1 mg/ml DNase I), and incubated at 37°C for 1 h. After digestion, cold Wash Medium was added to each tube to a total volume of 15 ml, the digested sample was filtered through a 70 μm strainer into a 50 ml centrifuge tube, and cold Wash Medium was added to a final volume of 50 ml. Samples were pelleted by centrifugation at 300 g for 5 min at 8°C, supernatants discarded, and cold Wash Medium added to a final volume of 15 ml. Resuspended material was transferred to a 15 ml centrifuge tube and the centrifugation, supernatant discard, and cell resuspension steps were repeated twice with cold Wash Medium and once with Basal Medium. After the last centrifugation step, cell pellets were mixed with Matrigel (Corning Biocoat) and seeded in 50 μl per well of prewarmed (37°C) 24-well plates. The volume of Matrigel was adjusted according to the number of cells. Each well-contained approximately 1,000 cells. After incubating the plate for 10 min at 37°C, until the mixture was solidified, 500 μl per well of Isolation Medium [Basal medium; 1:50 B27; 1:100 N2; 1 mM N-acetylcysteine; 100 μg/ml RSPO1; 10 mM nicotinamide; 10 nM human (Leu15)-gastrin I; 50 ng/ml human EGF; 100 ng/ml human FGF10; 25 ng/ml human HGF; 10 μm Forskolin; 5 μM A83-01; 25 ng/ml human Noggin; 200 μg/ml Wnt3a; 10 μM Y-27632] was added to overlay the droplet for the first 3–4 days. Then Isolation Medium was replaced with normal liver Expansion Medium (Isolation Medium without Noggin, Wnt-3a, or Y-27632). Medium was changed every 3–4 days. Organoids were visible within 7 days and ready for passage before day 14 of culture.

### Establishment of an Infection Model

A single isolated KPN colony was picked, then diluted with sterile normal saline, and measured using a McFarland turbidimeter to prepare 0.5 MCF (McFarland turbidity unit) bacterial solution. After gradient dilution, 50–100 μl solution was inoculated and streaked evenly on agar culture plates using a sterile spreader. Plates were stored for 20 min, until the solution penetrated into the medium, incubated in a 37°C incubator overnight, and counted the next day. Two maximum dilution gradients were chosen and three parallel experiments were conducted for each.

Bacterial solution (0.5 MCF) was added to the organoid culture medium (without P/S) at a ratio of 1:10. After incubating for 2 h at 37°C, fresh sterile medium was added to culture the organoids for 24 h. Culture supernatants were collected after centrifugation and stored at −80°C for subsequent experiments. To extract RNA from organoids, TRIzol (200 μl) was used to dissolve samples, which were mixed well and RNA extracted. For protein extraction, samples were incubated in 150 μl RIPA (Thermo Scientific) lysis buffer and protease inhibitor (100:1), dissolved and mixed, and proteins extracted.

### Bulk RNA Sequencing

A total of 1 μg RNA per sample was used for RNA sample preparation. Library construction was performed using an NEBNext Ultra^TM^ RNA Library Prep Kit, following the manufacturer's protocols. Index codes were added to attribute sequences to each sample. Libraries were pooled and sequenced on an Illumina Novaseq platform, and 150 bp paired-end reads were generated.

Raw data in fastq format were first processed using in-house Perl scripts. Clean data were obtained by removing reads containing adapter and poly-N sequences, as well as low-quality reads, from the raw data. Q20, Q30, and GC content values were calculated from the clean data, to ensure that downstream analyses were based on high-quality, clean data. Reference genome and gene model annotation files were downloaded directly from the genome website. Hisat2 v2.0.5 was used to build the index reference genome and align paired-end clean reads to the reference genome (27). FeatureCounts v1.5.0-p3 was used to count read numbers mapped to each gene (28). Fragments per kilobase per million mapped reads (FPKM) values were calculated for each gene, to correct for sequencing depth and gene length (29). Differential expression analysis was performed using the DESeq2 R package (1.16.1) (30). Differentially expressed genes (DEGs) were defined by a cut-off adjusted *p*-value of 0.05 and Log2 Fold-Change value of 1. Gene Ontology (GO) enrichment analysis, KEGG enrichment analysis, and Gene Set Enrichment Analysis (GSEA) of DEGs were implemented using the clusterProfiler R package (31). Gene counts of RNA-seq was uploaded separately as Supplementary Material.

### qPCR Analysis

Total RNA was extracted from organoids using TRIzol, then 1 μg was transcribed into cDNA using a reverse transcription kit (Takara, AK4601), according to the manufacturer's protocol. qPCR was performed according to the PCR amplification kit (Takara, AKA606). Reaction mixtures (10 μl) contained SYBR 5 μl, cDNA 2 μl, forward primer (5 μM) 0.5 μl, reverse primer (5 μM) 0.5 μl, and sterile ddH_2_O 2 μl. The reaction procedure was as follows: an initial 95°C (10 min), then 40 cycles of denaturation at 95°C for 5 s, annealing at 60°C for 30 s, and primer extension at 72°C for 35 s. Primer sequences for each gene are presented in Supplementary Table 5, and each sample was analyzed in duplicate. Relative RNA expression levels were calculated using the 2^−Δ*ΔCt*^ method, where ΔΔCt = experimental group ΔCt (Δ*Ct*_target gene_ – Δ*Ct*_GAPDH_) – control group ΔCt (Δ*Ct*_target gene_ – Δ*Ct*_GAPDH_).

### Enzyme-Linked Immunosorbent Assay (ELISA)

Organoid culture samples were obtained after infection, centrifuged at 1,000 g for 20 min, and stored at −80°C for future experiments. ELISA assay kits were applied to determine the levels of TGF-β1, IL-13, IL-33, and collagen type I alpha-1, according to the manufacturer's protocols (Cloud-Clone Corp., China). All samples were detected once using their corresponding kit with duplicate samples. Optical density (OD) was read at 450 nm for all indices, using a Varioskan LUX microplate reader (Thermo Scientific) with SkanIt Software 6.0.2.

### Western Blot Analysis

Organoids and culture supernatants were used to perform western blotting. Culture supernatants were centrifuged at 1,000 g for 20 min and supernatants were collected. Organoid proteins were extracted in RIPA buffer supplemented with protease inhibitors. Protein concentrations were determined using the BCA method (Beyotime, P0010S), and proteins denatured by heating at 95°C and mixed with 1 × loading buffer. Protein amounts and volumes were the same within groups. Samples were loaded on SDS-PAGE gels, electrophoresis was conducted for 1 h 40 min, and proteins were transferred to nitrocellulose membranes using a wet-transfer method for 1.5 h. Membranes were blocked in 5% skimmed milk for 1 h, and incubated overnight at 4°C with the following primary antibodies: IL-13 (Abcam, ab106732, 1:1,000), IL-33 (Abcam, ab54385, 1:1,000); TGF-β1 (Abcam, 215715, 1:1,000), and COL1A1 (CST, #72026S, 1:1,000). Organoid proteins levels were normalized to those of GAPDH (Proteintech, HRP-60004, 1:5,000). Culture supernatants were normalized by loading the same volume and concentration of samples, as determined by the BCA method. After washing with TBST buffer, membranes were incubated with HRP-conjugated secondary antibodies of corresponding species (anti-rabbit or anti-mouse) (1:5,000) for 1 h at room temperature, and proteins were visualized using an ECL kit on chemiluminescence apparatus. We used ImageJ (version 1.53C) for quantification of western blots.

### Statistical Analysis

All statistics were calculated using R program (V.3.6.2) and GraphPad Prism (version 8.0). Results are shown as mean ± SEM. A two-tailed unpaired Student's *t*-test was used for comparisons between two groups with normally distributed data and the Mann-Whitney test was used for comparisons of two groups with non-normally distributed data. The ADONIS test, a multivariate analysis of variance based on permutation, or a non-parametric MANOVA (Multivariate analysis of variance), was used to assess the significance of β-diversity. *P*-values <0.05 were considered statistically significant.

## Results

### Distinct Gut Microbiome Composition Before and After Kasai Procedure

**Figure 1:**
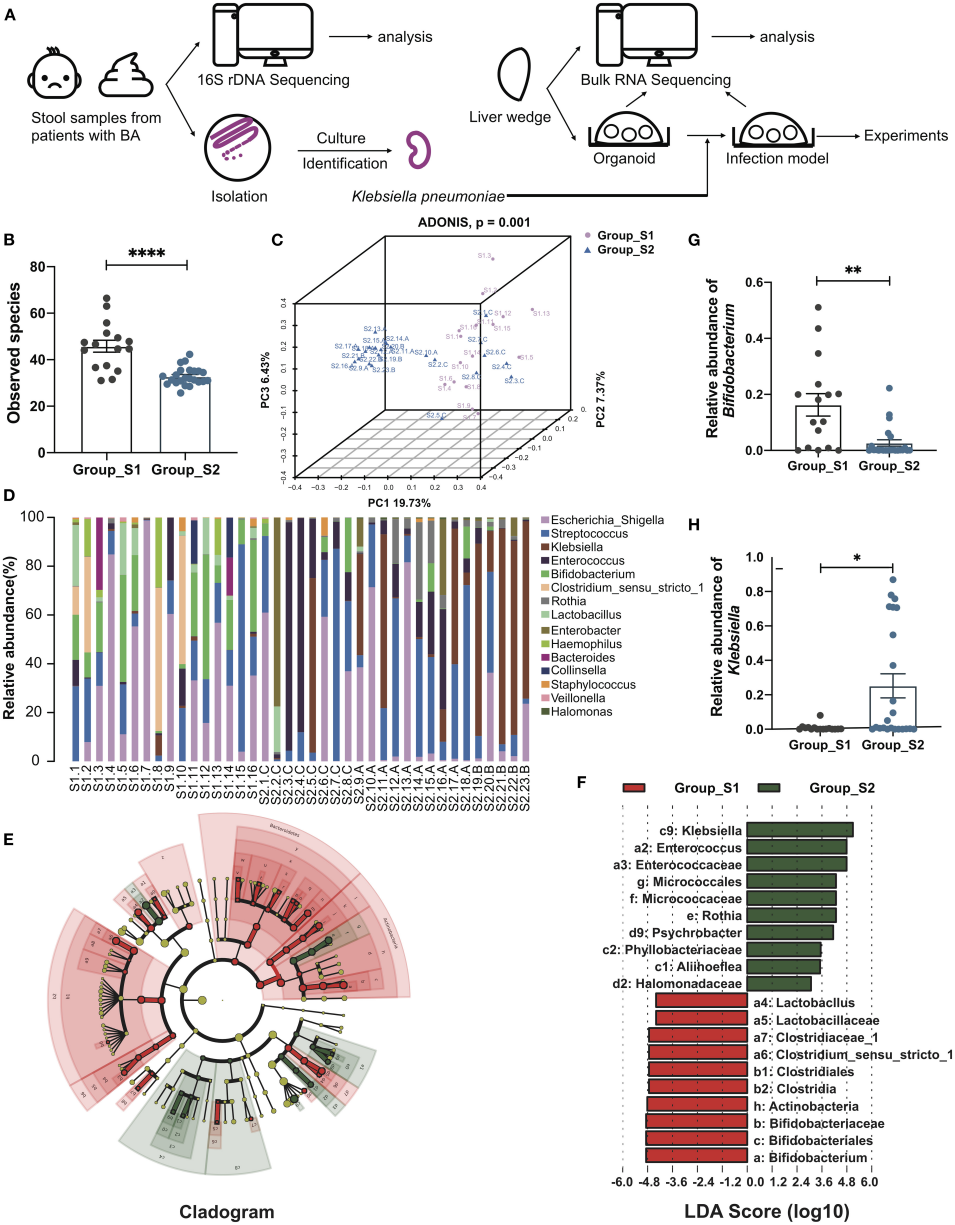
Distinct gut microbiome composition before and after Kasai surgery. **(A)** The whole workflow of this study. **(B)** Bar plot of observed species in patients before (Group_S1, *n* = 16) and after (Group_S2, *n* = 23) Kasai surgery. Statistical differences were analyzed by Mann-Whitney test. **(C)** Beta diversity PCoA plot of Binary Jaccard distances in patients before (Group_S1, *n* = 16) and after (Group_S2, *n* = 23) Kasai surgery. Each point represents a sample. Similar samples cluster together. There was a significant difference between the two groups (ADNOIS test). **(D)** Bar plot showing the relative abundance of the top 15 genera in each sample. **(E)** Cladogram of LEfSe analysis, showing the differential abundance between the two groups. Different colors represent different groups. Red nodes, microbiota with significantly higher abundance in Group_S1. Green nodes, microbiota with significantly higher abundance in Group_S2. Yellow nodes, microbiota with no significant difference between the two groups. Node size indicates relative abundance. Numbers are annotated in **(F)**. **(F)** Bar plot showing 10 differential taxa in each group ranked by LDA score. **(G,H)** Bar plots showing the relative abundance of *Bifidobacterium* and *Klebsiella* in both groups (Group_S1, *n* = 16; Group_S2, *n* = 23). The significance of differences was assessed by Mann-Whitney test. *p < 0.05; **p < 0.01; ****p < 0.0001.

The whole workflow was shown in Figure 1A. After raw data processing, quality control, and normalization, valid tags and OTUs were obtained for subsequent analyses (Supplementary Table 2). A total of 287 OTUs were obtained and the number of observed OTUs was significantly lower after than before the Kasai procedure (45.79 ± 2.56 before vs. 32.80 ± 0.80 after, *p* < 0.0001, Mann-Whitney test) (Figure 1B; Supplementary Table 3), indicating lower alpha diversity in post-Kasai samples. Decreased species richness was also confirmed by calculating Chao1, Shannon, and Simpson diversity indices, with significant *p*-values (Supplementary Figures S1A–C). A beta diversity principal coordinates analysis (PCoA) plot of Binary Jaccard distances was constructed using the OTUs and demonstrated a significant difference in microbial structural between the two groups (ADONIS test, *p* = 0.001) (Figure 1C). Based on taxonomic analysis of relative abundance, the bar plot and heatmap revealed differences in microbial composition before and after Kasai procedure at the genus level. *Klebsiella* were clearly enriched in Group_S2, while *Bifidobacterium* was enriched in Group_S1. Further, the composition diversity of Group_S1 was also higher than that of Group_S2 (Figure 1D; Supplementary Figure S1D). Linear discriminant analysis effect size was conducted to identify differences between the two groups and specifically the relative abundances of taxonomic categories. The top three most abundant species in Group_S1, *Bifidobacteriale, Bifidobacteriaceae*, and *Bifidobacterium*, all belonged to the genus, *Bifidobacterium* (*p* = 0.0023, Mann-Whitney test), while *Klebsiella* species (genus *Klebsiella*) were the most enriched in Group_S2 (*p* = 0.0160, Mann-Whitney test) (Figures 1E–H). In summary, these results show that gut microbiota biological diversity was significantly reduced, and the *Klebsiella* genus more abundant, in Group_S2, indicating alterations in microbial structure before and after Kasai procedure.

### Risk Factors Associated With Post-Kasai Cholangitis

**Figure 2:**
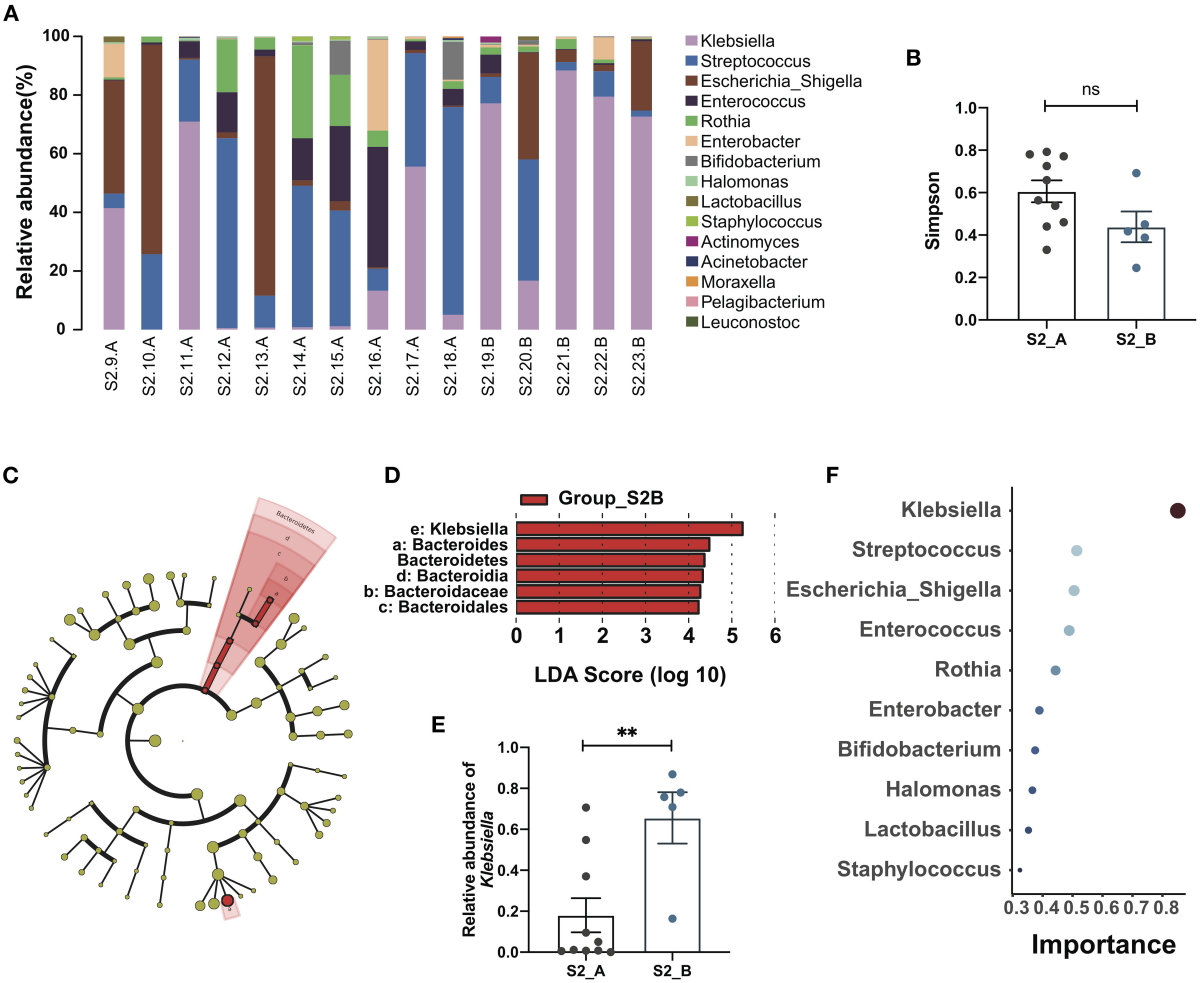
Gut microbiome changes and the importance of *Klebsiella* in post-Kasai cholangitis. **(A)** Bar plot showing the relative abundance of the top 15 genera in each sample. **(B)** Differences between Group S2_A (*n* = 10) and Group S2_B (*n* = 5). Simpson diversity index values were not statistically significant (Mann-Whitney test). **(C)** Cladogram of LEfSe analysis showing differential abundances between the two groups. Red nodes, species significantly differentially abundant in Group_S2B. Yellow nodes, microbiota that did not differ significantly between the two groups. Letters are annotated in **(D)**. **(D)** Bar plot showing the differential microbiota in Group_S2B ranked by LDA score, indicating that *Klebsiella* contributed the most to the difference between S2B and S2A. **(E)** Bar plot showing the relative abundance of *Klebsiella* in both groups (S2_A, *n* = 10; S2_B, *n* = 5). The significance of differences was determined by Mann-Whitney test. **(F)** Random Forest plot showing the top 10 genera ranked by the importance. The smaller a dot is, the higher its importance.**p < 0.01; ns, not significant.

According to follow-up data, samples in the Group_S2 were further divided into two subgroups, S2_A and S2_B, depending on whether the patient developed cholangitis within 1 month after discharge. A bar plot and heatmap of the genus-level microbial structures of the two groups showed the top 15 bacteria by relative abundance, and suggested a greater abundance of *Klebsiella* in the S2_B group, while the distribution of the genera in the S2_A group was more uniform (Figure 2A; Supplementary Figure S2A). Although alpha and beta diversity analysis did not reveal significant differences between the groups, they did demonstrate a different composition and lower species richness in group S2_B relative to S2_A (Figure 2B; Supplementary Figures S2B–E). Linear discriminant analysis effect size analysis revealed that *Klebsiella* made the largest contribution, with the highest linear discriminant analysis (LDA) score, indicating that this genus makes a significant contribution to the difference between the two groups. There was also a clear difference in relative abundance between these two groups (*p* = 0.0047, Mann-Whitney test) (Figures 2C–E). Next, we constructed a Random Forest model to predict Group_S2 subtypes with potential to predict post-Kasai cholangitis. In the Random Forest model for species importance, *Klebsiella* was ranked first (importance value 0.85, which is calculated by the algorithm, stands for the possibility of prediction), and was much higher than other genera (Figure 2F). Therefore, *Klebsiella* can be used as a factor that predicts the occurrence of cholangitis after Kasai procedure. These data suggest that biliary drainage by Kasai surgery and post-operative use of antibiotics may inhibit the growth of various bacteria, while *Klebsiella* may be relatively tolerant to this inhibition, and could be the main cause of post-operative cholangitis.

### Organoids Could Be an Appropriate Model for BA

**Figure 3:**
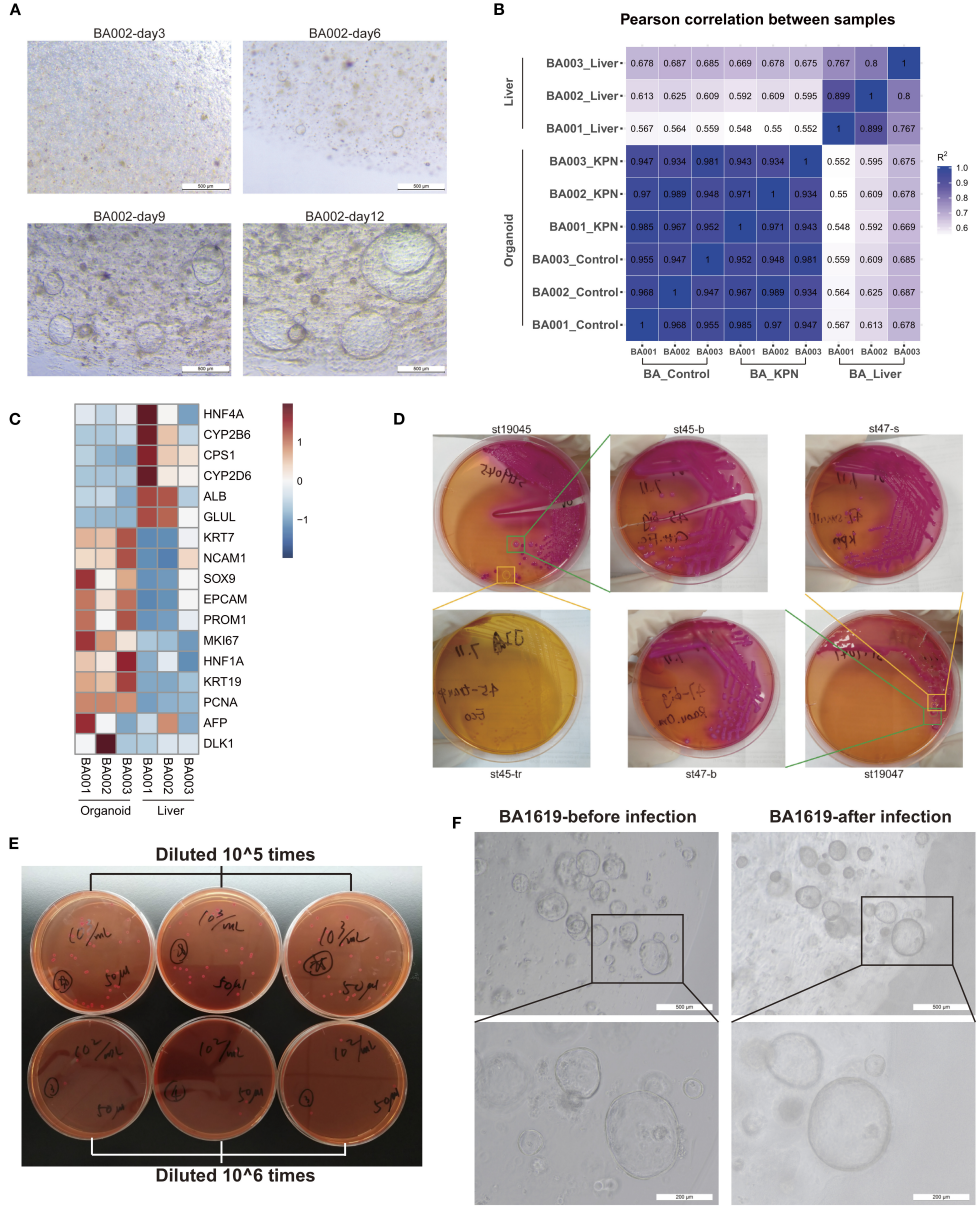
Organoids may be an appropriate model for BA and post-Kasai cholangitis. **(A)** Culture of BA liver organoids. Small organoids began to appear during days 4–6 of culture and grew into large, mature, spherical organoids by days 10–12. **(B)** Pearson's correlation test of three liver tissues, corresponding to organoid control and KPN samples. High correlation was observed among organoid samples. **(C)** Heatmap of marker gene expression in tissue and organoid samples, indicating that organoids expressed higher levels of progenitor markers, while tissue samples expressed higher levels of mature hepatocyte markers. **(D)** Part of the bacterial isolation and passaging process. KPN colonies appeared as large, mucoid, and pink on MacConkey Agar. **(E)** Bacteria counting process. The concentration of 0.5 MCF KPN bacterial solution was approximately 6.36 × 10^7^ CFU/ml. **(F)** The same organoids were observed before and after infection with KPN. Organoids presented crenulation and deformation with thicker cell layers after infection.

Liver tissue samples were collected from children with type III BA during surgery, and liver organoids were established following the protocols described in the Methods section. Small organoids began to appear after 4–6 days of culture and grew into large mature organoids, shaped as spheres, by 10–12 days (Figure 3A). We performed RNA-seq using three liver tissue and three organoid model samples and conducted Pearson's correlation tests on all samples. The results indicated stable and homogeneous characteristics within organoid models, while liver samples showed substantial heterogeneity among individuals (Figure 3B). Next, we compared the expression levels of some marker genes (Figure 3C). Compared with liver tissues, BA organoids expressed higher levels of the proliferation genes, *MKI67* (organoid vs. liver 39.72 vs. 6.70, *p* = 0.0105, Student's *t*-test) and *PCNA* (organoid vs. liver 268.41 vs. 62.64, *p* < 0.0001, Student's *t*-test). Genes representing hepatic duct progenitor cells, including *KRT19* (organoid vs. liver 2563.77 vs. 111.63, *p* = 0.0023, Student's *t*-test), *KRT7* (organoid vs. liver 497.78 vs. 96.40, *p* = 0.0183, Student's *t*-test), and the early hepatic stem marker genes, *EPCAM* (organoid vs. liver 278.72 vs. 76.10, *p* = 0.0269, Student's *t*-test), *PROM1*, and *HNF1A*, were all upregulated in organoids. Liver tissues expressed higher levels of mature hepatocyte cell markers, including *ALB, CPS1*, and *GLUL*, among others. Therefore, BA organoids simultaneously expressed the characteristics of hepatic progenitor cells, hepatocytes, and hepatic duct progenitor cells, with strong proliferation ability. More importantly, as they are derived from patients, organoids can mimic the disease states and genetic features of patients.

### Post-Kasai Cholangitis Model Established by Infection With *Klebsiella*

The migration of dominant flora may lead to the onset of cholangitis after Kasai procedure. Therefore, we decided to use patient-derived specific bacteria to infect patient-derived organoids, to establish an *in vitro* infection model of post-Kasai cholangitis. Stool samples were collected and inoculated on MacConkey Agar on the same day. Mass spectrometry identification was conducted on the second day when isolated colonies formed (Supplementary Table 4). Next, we passaged cultures 2–3 times, until the strains were purer and more stable. The bacterial isolation and passage process is illustrated in Figure 3D. Based on the results of 16s rDNA sequencing, the dominant genus in children with BA post-Kasai procedure was *Klebsiella*, and the identification procedure described above showed that the strain, *Klebsiella pneumoniae subsp. pneumoniae* (KPN) *9295_1 CHB*, comprised the highest proportion. Strain No. St53, which had the highest score, was selected for subsequent experiments. Normal saline was used to generate 0.5 MCF bacterial solutions, which were diluted 10^5^ and 10^6^ times, and smeared on culture plates to count bacterial colonies. The concentration of 0.5 MCF bacterial solution was approximately 6.36 × 10^7^ CFU/ml (Figure 3E). After infection for 2 h and culture with fresh medium for 24 h, the organoids presented with crenulation and deformation, with thicker cell layers, which displayed signs similar to aging and apoptosis (Figure 3F). The culture medium was turbid and turned yellow, typical of bacterial contamination. Bulk RNA-seq was also conducted on three infected organoid samples.

### Transcriptome Shift After Infection in BA Organoids

**Figure 4:**
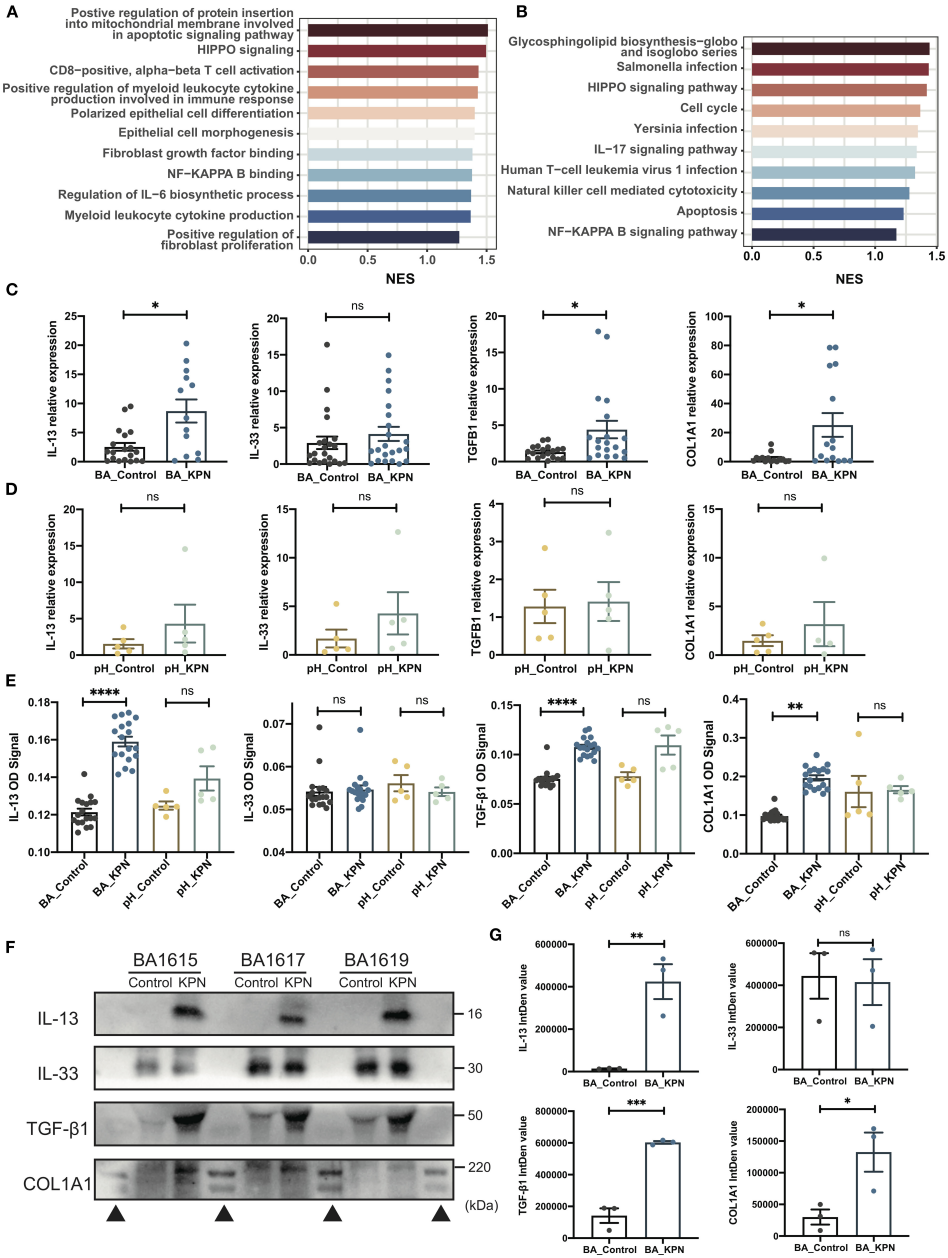
Preliminary study of the mechanism underlying fibrosis in post-Kasai cholangitis. **(A,B)** Enriched pathways determined by GO and KEGG analysis presented as bar plots, including infection-, immune-, and fibrosis-associated pathways. Length indicates the normalized enrichment score (NES). Color (blue to red) indicates score (lower to higher). **(C)** qPCR analysis of expression of the indicated genes in BA organoids from the infection and control groups (*n* = 23). The significance of differences was analyzed by Mann-Whitney test. **(D)** qPCR analysis of expression of the indicated genes in pH organoids from the control and infection groups (*n* = 5). The significance of differences was analyzed by Mann-Whitney test. **(E)** Target protein levels in BA organoid culture supernatants were measured by ELISA, and OD signal levels compared (BA_Control, *n* = 23; BA_KPN, *n* = 23; pH_Control, *n* = 5; pH_KPN, *n* = 5). **(F)** Western blot analysis of target proteins in BA organoid culture supernatants. Three samples (BA1615, BA1617, BA1619) were included. **(G)** Integrity density (IntDen) of western blots measured using ImageJ, and analyzed using the Mann-Whitney test. *p < 0.05; **p < 0.01; ***p < 0.001; ****p < 0.0001; ns, not significant; ▴, markers.

To investigate transcriptome changes after organoid infection, we used the DESeq2 algorithm to extract differentially expressed genes, ranked them according to average Log2 fold-change values, and used GSEA to identify pathways enriched during infection, similar to the detection of pathways involved in post-Kasai cholangitis. We screened out the most relevant gene sets enriched in KEGG (Kyoto Encyclopedia of Genes and Genomes) and GO analyses (Figures 4A,B). In the KEGG gene set, the cholangitis model was clearly enriched for pathways associated with infection, consistent with the results of GO analysis. Other enriched pathways included inflammation, immune activation, and cytokine production. Moreover, analysis of epithelial morphogenesis and apoptosis demonstrated injury to duct epithelial cells, consistent with our experimental observations. Fibrosis-associated pathways may support the increased fibrosis in patients with post-Kasai cholangitis.

### Fibrosis-Related Genes Are Overexpressed in the Post-Kasai Cholangitis Organoid Model

The expression of type 2 cytokines is reported to promote cholangiocyte proliferation and hepatic fibrosis in BA (32), and the IL-33–IL-13 axis was correlated with liver fibrosis in patients with BA in our former study (33). Therefore, we investigated whether this pathway is involved in progressive fibrosis after cholangitis by analyzing the expression of *IL33, IL13, TGFB1*, and *COL1A1*. There was no significant difference in *IL33* levels, while those of *IL13, TGFB1*, and *COL1A1* were significantly increased after infection (Figure 4C). When para-hepatoblastoma (pH) normal liver-derived organoids were subjected to the same intervention, none of these genes were upregulated at the mRNA level (Figure 4D). To further test those targets at the protein level, we first performed ELISAs, which demonstrated substantial increases in IL-13, TGF-β1, and type I collagen in BA organoids after KPN infection (Figure 4E). Western blotting analysis of BA organoid proteins revealed that only TGF-β1 showed a rising trend, while no images were generated for the other proteins (Figure 4F; Supplementary Figures S3D,E), likely because all the investigated proteins were secreted. Therefore, we performed western blots using BA culture supernatants; the results were consistent with those of ELISAs, with expression levels of IL-13, TGFβ1, and type I collagen showing remarkable increases after infected with KPN (Figure 4). Again, in infected pH organoids, ELISA showed no substantial increase in the four target proteins.

## Discussion

The specific etiology and mechanism underlying post-Kasai cholangitis remain unclear; however, its occurrence influences the short- and long-term prognosis of children with BA. In recent years, the theory that intestinal flora migration contributes to development of post-Kasai cholangitis has become generally accepted. The Kasai procedure reconstructs the bile drainage system by connecting the biliary system with the intestine, thereby increasing the chance for intestinal flora migration and direct invasion causing infection. The fasting of children during the perioperative period and surgical operations to the gut also weaken the intestinal barrier function, increasing the possibility of intestinal flora migration to distant organs through the intestinal mucosa. A study involving 16S rDNA sequencing, isolation, and culture of blood samples from children with BA showed that the positive rate of bacterial DNA in children with cholangitis after surgery was higher than that in children without cholangitis. The bacteria detected by 16S rDNA were mainly opportunistic and pathogenic bacteria from the intestine, including *E. coli, KPN, Shigella flexneri*, and *Enterobacteriaceae bacterium*. In addition, the positive rate of bacterial DNA is significantly related to end-stage liver disease score, procalcitonin, C-reactive protein, and heart rate (34). These findings indicate that migration of the gut microbiome may be important in the pathogenesis of post-Kasai cholangitis. Therefore, we conducted a study of stool samples, to explore the bacterial characteristics, and investigate the relationship between gut microbiome alterations and the occurrence of cholangitis.

In this study, we found that gut microbiome diversity was significantly reduced in patients with BA who underwent Kasai surgery, with a tendency toward a simplified structure. At the genus level, the dominant genus changed from *Bifidobacterium* to *Klebsiella*. Reconstruction of bile drainage and the intestine using the Kasai procedure to reinduce bile acid flow may kill many bacteria (35), and long-term antibiotics use may also contribute to these alterations. All patients in this study received the same antibiotic therapy in the hospital. Intravenous prophylactic antibiotics are currently the routine treatment for BA after Kasai. Patients receive intravenous antibiotics for approximately 2 weeks after the Kasai procedure and then transfer to oral prophylactic antibiotics for 6 months. Although antibiotics are primarily used to kill suspected pathogens, they also directly or indirectly threaten intestinal commensal bacteria and beneficial flora to some extent (36, 37). This may directly influence the abundance of beneficial microbes, such as those of the genus *Bifidobacterium*. Antibiotics can affect nutrient absorption and energy supply in intestinal epithelial cells, cause intestinal cell apoptosis, weaken the intestinal barrier function, promote the migration of bacteria and their products, and finally increase the risk of disease, by reducing the flora beneficial to metabolism and disrupting microbiome homeostasis (38).

Despite the long-term preventive use of antibiotics following Kasai surgery, almost half of children develop cholangitis while taking the medication. Therefore, we further explored the characteristics of intestinal flora in children with and without cholangitis. Our results showed that, compared with children who did not develop post-Kasai cholangitis within 1 month, there was no significant difference in gut microbiome diversity in children with early-onset cholangitis; however, the structure was simplified and significantly dominated by the genus, *Klebsiella*. Regardless of the relative abundance and importance of species, the genus *Klebsiella* was significantly more abundant than other genera, indicating that *Klebsiella* may be relatively resistant to prophylactic antibiotics, and the main pathogen causing post-Kasai cholangitis. Long-term use of antibiotics may allow some antibiotic-resistant pathogenic bacteria and opportunistic pathogens to multiply (36); therefore, patient intestinal flora should be monitored following Kasai surgery, like the routine stool culture, and their specific microbiome status evaluated, to prevent or treat cholangitis more precisely. Moreover, individualized medication plans for patients could also be developed, with the aim of preventing the emergence of drug-resistant bacteria. Other than using antibiotics as routine medication to prevent post-Kasai cholangitis, supportive treatments, such as probiotics and post-operative nutrition management, could also have preventive effects. *Lactobacillus rhamnosus* can reduce the incidence of cholangitis following Kasai surgery by competitively inhibiting other intestinal pathogens (39).

Based on our data we consider *Klebsiella* a key pathogen involved in post-Kasai cholangitis. We used the strain, KPN CHB, isolated from patient stool, as a pathogen for use in our infection model. Due to the lack of *in vivo* or *in vitro* post-Kasai cholangitis models, we generated an organoid model, as these are able to model disease *in vitro* by closely mimicking *in vivo* conditions (40). In recent years, organoid technology has become a powerful tool for disease modeling that maintains cells in a near-native state (40). Organoids are directly derived from patient tissues, which can contain all the cellular components of the original microenvironment, as well as having the patient's genetic characteristics (41). Organoids can retain the properties of stem cells, which was also reflected in our study, where our organoids expressed stem cell marker genes. Furthermore, intra-patient heterogeneity was preserved to a certain extent, whereas the interpatient heterogeneity was less than that observed in liver samples. These advantages make organoids stable, effective model systems.

After infection with KPN, bulk RNA-seq revealed a transcriptome shift toward inflammation and fibrosis, which was also supported by GSEA and consistent with patient clinical manifestations. We also conducted a preliminary investigation of the mechanism underlying fibrosis. In patients with BA, the IL-33/IL-13 pathway was correlated with liver fibrosis progression, in which IL-33 is secreted and binds ST2 receptors on mast cells to release IL-13/TGF-β1, which mediate the process of fibrosis (33). In our research, IL-33 was not significantly upregulated, whereas IL-13, TGF-β1, and type I collagen were highly expressed after infection, indicating that there are alternative pathways that induce overexpression of IL-13 and TGF-β1 and mediate liver fibrosis in BA.

IL-13 is a pro-inflammatory cytokine, which is mainly produced by Th2 lymphocytes, epithelial cells, innate lymphoid cells-2, macrophages, and less strongly by mast cells, eosinophils, basophils, CD8^+^ Th cells, and natural killer cells. It is established that IL-13 has a role in allergic inflammation (42). Further, a relationship between IL-13 and fibrosis has also been reported in many chronic infectious and autoimmune diseases (43), which is reported to signal through the IL-13α2 receptor to induce TGF-β1 production as a central mediator of fibrosis (43–45).

After KPN infection, *IL13, TGFB1, COL1A1* were substantially upregulated at the mRNA and protein levels in the BA group, indicating a type II immune response. One point that deserves further discussion is that most bacterial infections cause a type I immune response, whereas the type II cytokine, IL-13, was overexpressed in our model. Further, qPCR showed no significant differences in *IL1B* or *IL8* levels before and after infection (Supplementary Figures S3A,B). During infection, T cells appear to gradually lose the ability to secrete pro-inflammatory cytokines, and switch from a type I to a type II response as they mature after multiple cell divisions. In addition, severe systemic stress, immunosuppression, or overwhelming microbial inoculation cause type II responses (46), which may explain our findings. Moreover, IL-13 can activate monocyte cell lines, consistent with our GO analysis, and inhibit the production of inflammatory cytokines (such as IL-1β, IL-8, etc.) (47). In patients with BA, whose baseline expression of IL-13 is higher than that in healthy controls, the preexisting higher levels of IL-13 inhibit Th1 polarization, to suppress the type I immune response (48). The preexisting type II response alters the post-infection kinetics of inflammation, as also reported in allergic airway inflammation (49). This may explain why there were no significant differences in the pH group, which had lower preexisting IL-13 expression levels; however, there was a slightly rising trend in the pH group after infection, and experiments with a larger sample size are warranted to test this hypothesis. Overall, our results indicate that infections may enhance IL-13/TGFβ1 pathway-mediated fibrosis.

In addition to IL-13 signaling, cell death and apoptosis may be other core pathways involved in fibrosis. GSEA suggested a potential contribution of apoptosis, while qPCR analysis of *KRT19* expression indicated a slight decrease, and the expression of apoptosis-related genes (*CASP3, CASP9, BAX*) increased (Supplementary Figures S3C,F). Regardless of the apoptosis of epithelial or endothelial cells, local tissue damage, and pervasive cell death are believed to be crucial drivers of fibrosis. As well as IL-4 and IL-13 signaling, engagement of apoptotic cell sensors is also necessary to fully activate tissue repair macrophages and promote fibrosis (50). Studies have shown that apoptosis has been identified as a potential initiator and propagator of fibrosis in complex ways. On one hand, immune cells, including macrophages, neutrophils, and other leukocytes, are stimulated to secrete factors that contribute to fibrosis after apoptotic cells are engulfed. On the other hand, apoptosis cells are also possible to paracrine signal resulting in fibrosis (51).

In summary, our work revealed alterations in the gut microbiota following Kasai surgery and highlights a characteristic microbiome in patients who went on to develop post-Kasai cholangitis. The genus, *Klebsiella*, may be a key factor that can predispose to cholangitis. We isolated a specific strain, KPN, and used it to establish an organoid model of post-Kasai cholangitis and conduct preliminary experiments to determine an underlying mechanism mediated by the IL-13/TGF-β1 pathway. Together, our findings suggest that gut microbiota are important in the pathogenesis of post-Kasai cholangitis, and may provide insights to drive further research.

## Data Availability Statement

The datasets presented in this study can be found in online repositories. The names of the repository/repositories and accession number(s) can be found at: https://www.ncbi.nlm.nih.gov/bioproject/PRJNA738467.

## Ethics Statement

The studies involving human participants were reviewed and approved by the Ethics Committee of the Children's Hospital of Fudan University. Written informed consent to participate in this study was provided by the participants' legal guardian/next of kin.

## Author Contributions

LM and JL conceived and designed this study, performed the experiment, analyzed the data, and wrote the manuscript. JW and MD participated in discussion of the results and offered scientific advice. SZ provided part of the samples. YH, ZS, RD, GC, and SZ supervised the study. SZ reviewed and revised this manuscript. All authors read and approved the final paper.

## Funding

This study received financial support from Clinical Research Plan of SHDC (No. SHDC2020CR2009A), Shanghai Municipal Key Clinical Specialty (No. shslczdzk05703), National Natural Science Foundation of China (Nos. 81770519, 81771633, 81873545, 81974059, and 81960101), the Science Foundation of Shanghai (Nos. 18411969100 and 19ZR1406600), and Children's National Medical Center (Nos. EK1125180104, EKYY20180204, EK112520180211, and EK112520180310).

## Conflict of Interest

The authors declare that the research was conducted in the absence of any commercial or financial relationships that could be construed as a potential conflict of interest.

## Publisher's Note

All claims expressed in this article are solely those of the authors and do not necessarily represent those of their affiliated organizations, or those of the publisher, the editors and the reviewers. Any product that may be evaluated in this article, or claim that may be made by its manufacturer, is not guaranteed or endorsed by the publisher.
