# Supplementary material for: Characteristics of the Gut Microbiome and IL-13/TGF-β1 Mediated Fibrosis in Post-Kasai Cholangitis of Biliary Atresia
Source: Front Pediatr. 2021 Nov 8;9:751204. doi: 10.3389/fped.2021.751204 (PMC8630618; doi:10.3389/fped.2021.751204)
Supplement: Supplementary file 1 [file Data_Sheet_1.docx]

***Supplementary materials***

1. **Supplementary Methods**
   1. **Diagnosis criteria**

Participants were diagnosed with type III biliary atresia (BA) by intraoperative cholangiogram and liver biopsy pathology. The inclusion criteria were as follows: infants with gestational age at birth ≥ 36 weeks and obstructive jaundice diagnosed with type III BA, according to the BA phenotype classification in our hospital. Patients with other digestive system diseases or serious diseases of other systems were excluded.

Children diagnosed with post-Kasai cholangitis met the following two conditions simultaneously: (1) Excluding fever caused by other infections, body temperature > 38°C, or laboratory examination showing C-reactive protein exceeding the normal level (> 8 mg/L); and (2) Stool color became lighter or clinical symptoms of jaundice increased, and liver function test showing total bilirubin (TB) levels were higher than the most recent previous result.

Note: According to the routine BA diagnosis and treatment protocol at our clinical center, all children with BA were hospitalized for 14 days after Kasai surgery, and intravenous antibiotics (cefoperazone-sulbactam + ornidazole) administered prophylactically during hospitalization. After discharge from hospital on the 14th day, patients were prescribed oral prophylactic antibiotics (alternate use of cefaclor/sulfamethoxazole-trimethoprim) until 6 months after surgery.

This study was approved by the Ethics Committee of the Children’s Hospital of Fudan University, and informed consent was obtained from the legal guardians of each patient.

1. **Supplementary Tables**
   1. **Supplementary Table 1 Group information of 39 stool samples**

| Group | Sub-group | Sample No. | Sample size | Time of sample collection | Episodes of cholangitis within 1 month after discharged (Day 14~ Day 44) |
| --- | --- | --- | --- | --- | --- |
| Group_S1 | - | S1.1, S1.2,…, S1.16 | 16 | Day -1 | - |
| Group_S2 | S2_C | S2.1.C, S2.2.C,…, S2.8.C | 8 | Day 14 | Unknown |
|  | S2_A | S2.9.A, S2.10.A,…, S2.18.A | 10 |  | 0 |
|  | S2_B | S2.19.B, S2.20.B,…, S2.23.B | 5 |  | ≥1 episode |

Note: Day -1 is one day before Kasai, Day 0 is the day received Kasai, and so on.

- 1. **Supplementary Table 2 Valid tags and OTUs of all samples**

| Sample ID | Clean tags | Valid tags | Valid tags% | Valid tags mean Length | OTUs counts |
| --- | --- | --- | --- | --- | --- |
| S1.1 | 41885 | 38007 | 90.74% | 435.16 | 67 |
| S1.2 | 38770 | 35299 | 91.05% | 429.28 | 35 |
| S1.3 | 41263 | 38226 | 92.64% | 435.33 | 35 |
| S1.4 | 41723 | 40154 | 96.24% | 440.6 | 52 |
| S1.5 | 40076 | 35862 | 89.48% | 432.73 | 68 |
| S1.6 | 38818 | 35649 | 91.84% | 437.04 | 48 |
| S1.7 | 38003 | 37070 | 97.54% | 440.88 | 54 |
| S1.8 | 33532 | 31143 | 92.88% | 426.11 | 58 |
| S1.9 | 38767 | 36675 | 94.60% | 440.96 | 30 |
| S1.10 | 38553 | 35773 | 92.79% | 433.56 | 36 |
| S1.11 | 41067 | 37083 | 90.30% | 432.94 | 45 |
| S1.12 | 40003 | 36598 | 91.49% | 430.8 | 33 |
| S1.13 | 25936 | 23701 | 91.38% | 439.68 | 47 |
| S1.14 | 39300 | 36064 | 91.77% | 432.07 | 48 |
| S1.15 | 38064 | 36634 | 96.24% | 438.97 | 42 |
| S1.16 | 29712 | 26980 | 90.81% | 432.85 | 48 |
| S2.1.C | 38209 | 35601 | 93.17% | 439.75 | 37 |
| S2.2.C | 39310 | 37218 | 94.68% | 440.88 | 31 |
| S2.3.C | 38506 | 37232 | 96.69% | 440.78 | 42 |
| S2.4.C | 38685 | 36935 | 95.48% | 440.92 | 35 |
| S2.5.C | 39540 | 37441 | 94.69% | 440.9 | 38 |
| S2.6.C | 37702 | 35476 | 94.10% | 439.89 | 43 |
| S2.7.C | 37995 | 36755 | 96.74% | 440.64 | 30 |
| S2.8.C | 34696 | 32220 | 92.86% | 436.47 | 33 |
| S2.9.A | 57661 | 55795 | 96.76% | 463.74 | 35 |
| S2.10.A | 120311 | 113124 | 94.03% | 463.54 | 28 |
| S2.11.A | 101409 | 98967 | 97.59% | 464.73 | 31 |
| S2.12.A | 110967 | 108649 | 97.91% | 460.35 | 34 |
| S2.13.A | 98295 | 96466 | 98.14% | 464.13 | 31 |
| S2.14.A | 113362 | 110784 | 97.73% | 458.71 | 34 |
| S2.15.A | 111004 | 108013 | 97.31% | 459.33 | 39 |
| S2.16.A | 109501 | 105877 | 96.69% | 463.07 | 33 |
| S2.17.A | 119660 | 114057 | 95.32% | 464.76 | 29 |
| S2.18.A | 107945 | 106265 | 98.44% | 461.91 | 31 |
| S2.19.B | 92999 | 90419 | 97.23% | 464.28 | 30 |
| S2.20.B | 103990 | 100718 | 96.85% | 463.14 | 31 |
| S2.21.B | 78669 | 76986 | 97.86% | 464.25 | 35 |
| S2.22.B | 86060 | 83490 | 97.01% | 464.51 | 31 |
| S2.23.B | 89344 | 87644 | 98.10% | 464.75 | 26 |

- 1. **Supplementary Table 3 Index of alpha diversity in all samples**

| Samples | group | Chao1 | Observed species | Shannon | Simpson |
| --- | --- | --- | --- | --- | --- |
| S1.1 | Group_S1 | 94.0048016 | 63 | 3.15305962 | 0.84652128 |
| S1.2 | Group_S1 | 49.2083333 | 31.5 | 2.42993695 | 0.7648431 |
| S1.3 | Group_S1 | 55.67 | 36.6 | 2.41538902 | 0.76589597 |
| S1.4 | Group_S1 | 75.3729762 | 44.6 | 1.07896365 | 0.28989115 |
| S1.5 | Group_S1 | 112.739924 | 66.4 | 3.1618194 | 0.85008731 |
| S1.6 | Group_S1 | 69.6709524 | 44.1 | 2.70999005 | 0.72712134 |
| S1.7 | Group_S1 | 97.9018615 | 49.4 | 0.1896367 | 0.03263836 |
| S1.8 | Group_S1 | 96.0315476 | 55.8 | 1.81251911 | 0.59500878 |
| S1.9 | Group_S1 | 71.87 | 31 | 1.39024036 | 0.55083679 |
| S1.10 | Group_S1 | 57.115 | 37.4 | 2.18968827 | 0.69068331 |
| S1.11 | Group_S1 | 87.1116667 | 47.4 | 3.02006687 | 0.83275666 |
| S1.12 | Group_S1 | 58.7 | 35.1 | 2.03918309 | 0.67763057 |
| S1.13 | Group_S1 | 100.450833 | 51.4 | 2.26499085 | 0.65161576 |
| S1.14 | Group_S1 | 57.075 | 46.6 | 2.84538819 | 0.8159195 |
| S1.15 | Group_S1 | 82.7640476 | 44.6 | 1.22115522 | 0.35587666 |
| S1.16 | Group_S1 | 95.295 | 47.8 | 2.66258183 | 0.7531681 |
| S2.1.C | Group_S2 | 67.4890476 | 35.1 | 1.61319103 | 0.5583163 |
| S2.2.C | Group_S2 | 47.5566667 | 29.5 | 2.03283494 | 0.66666464 |
| S2.3.C | Group_S2 | 104.483333 | 39.4 | 0.47335343 | 0.12784935 |
| S2.4.C | Group_S2 | 67.155 | 35.5 | 0.59460765 | 0.21799174 |
| S2.5.C | Group_S2 | 72.3121429 | 38.8 | 1.17154743 | 0.43858315 |
| S2.6.C | Group_S2 | 77.5536111 | 42.3 | 1.90140321 | 0.60124344 |
| S2.7.C | Group_S2 | 66.0116667 | 30.9 | 0.90158304 | 0.28095557 |
| S2.8.C | Group_S2 | 65.6359524 | 34.5 | 2.09493628 | 0.73128346 |
| S2.9.A | Group_S2 | 52.2190476 | 35.4 | 2.32278336 | 0.7261697 |
| S2.10.A | Group_S2 | 40.7785714 | 30.1 | 1.20083242 | 0.43961122 |
| S2.11.A | Group_S2 | 41.3392857 | 33.4 | 1.36526565 | 0.45742733 |
| S2.12.A | Group_S2 | 38.175 | 32.9 | 2.13734284 | 0.65806328 |
| S2.13.A | Group_S2 | 38.8 | 28.5 | 1.20961332 | 0.33266679 |
| S2.14.A | Group_S2 | 35.1383333 | 31.9 | 2.54387008 | 0.77981722 |
| S2.15.A | Group_S2 | 41.25 | 35.1 | 2.56778122 | 0.77190553 |
| S2.16.A | Group_S2 | 39.8857143 | 31.3 | 2.63806229 | 0.79147954 |
| S2.17.A | Group_S2 | 34.1866667 | 25.7 | 1.56070468 | 0.56523727 |
| S2.18.A | Group_S2 | 34.3166667 | 30.4 | 1.86042869 | 0.53819076 |
| S2.19.B | Group_S2 | 35.6916667 | 31 | 1.57014816 | 0.41480102 |
| S2.20.B | Group_S2 | 44.1116667 | 32 | 2.08157787 | 0.69316394 |
| S2.21.B | Group_S2 | 40.5133333 | 31.3 | 0.95322049 | 0.24475929 |
| S2.22.B | Group_S2 | 36.1433333 | 30.6 | 1.40376225 | 0.38752206 |
| S2.23.B | Group_S2 | 36.1283333 | 28.8 | 1.29611225 | 0.45067896 |

- 1. **Supplementary Table 4 Isolated bacteria classification results**

| Sample No. | Strain | Organism | Score Value | NCBI Identifier |
| --- | --- | --- | --- | --- |
| st19044 | st44-s | KPN-9295_1 CHB | 2.108 | 72407 |
| st19044 | st44-b | KPN-DSM 16358T DSM | 2.073 | 574 |
| st19045 | st45-b | KPN-37924 PFM | 1.909 | 573 |
| st19045 | st45-tr | Ecoli-MB11464_1 CHB | 2.327 | 562 |
| st19047 | st47-s | KPN-37924 PFM | 1.68 | 573 |
| st19047 | st47-b | *Raoultella planticola* | 2.183 | 575 |
| st19048 | st48 | *Klebsiella oxytoca* | 2.282 | 571 |
| st19049 | st49 | Ecoli-DSM 682 DSM | 2.294 | 562 |
| st19050 | st50 | Ecoli-DSM 682 DSM | 2.491 | 562 |
| st19051 | st51-l | KPN-9295_1 CHB | 2.143 | 72407 |
| st19051 | st51-d | *Citrobacter freundii* | 2.159 | 546 |
| st19052 | st52 | KPN-9295_1 CHB | 2.142 | 72407 |
| st19053 | st53 | KPN-9295_1 CHB | 2.325 | 72407 |
| st19054 | st54 | Ecoli-DH5alpha BRL | 2.246 | 562 |
| st19055 | st55-d | *Klebsiella oxytoca* | 1.982 | 571 |
| st19055 | st55-l | Ecoli-MB11464_1 CHB | 2.117 | 562 |
| st19056 | st56 | Ecoli-DH5alpha BRL | 1.917 | 562 |

KPN-9295_1 CHB, *Klebsiella pneumoniae subsp. pneumoniae 9295_1 CHB*; KPN-DSM 16358T DSM, *Klebsiella pneumoniae subsp. ozaenae DSM 16358T DSM*; KPN-37924 PFM, *Klebsiella pneumoniae 37924 PFM;* Ecoli-MB11464_1 CHB, *Escherichia coli MB11464_1 CHB*; Ecoli-DSM 682 DSM, *Escherichia coli DSM 682 DSM*; Ecoli-DH5alpha BRL, *Escherichia coli DH5alpha BRL*.

- 1. **Supplementary Table 5 Primers used in qPCR assays**

| Primers | Forward sequence | Reverse sequence |
| --- | --- | --- |
| *GAPDH* | GGGGAAGGTGAAGGTCGGAG | CCTGGAAGATGGTGATGGGA |
| *ACTA2* | CTATGCCTCTGGACGCACAACT | CAGATCCAGACGCATGATGGCA |
| *COL1A1* | GATTCCCTGGACCTAAAGGTGC | AGCCTCTCCATCTTTGCCAGCA |
| *IL13* | ACGGTCATTGCTCTCACTTGCC | CTGTCAGGTTGATGCTCCATACC |
| *IL33* | TTGGCATGCAACCAGAAGTC | CCTGTCAACAGCAGTCTACT |
| *KRT19* | AGCTAGAGGTGAAGATCCGCGA | GCAGGACAATCCTGGAGTTCTC |
| *TGFB1* | CAATTCCTGGCGATACCTCAG | GCACAACTCCGGTGACATCAA |

- 1. **Supplementary Table 6 Antibodies used in this study**

| Antibody | Cat NO. | Manufacturer |
| --- | --- | --- |
| GAPDH | HRP-60004 | Proteintech |
| COL1A1 | #72026S | Cell Signaling Technology |
| IL-13 | ab106732 | Abcam |
| IL-33 | ab54385 | Abcam |
| TGFB1 | 215715 | Abcam |

1. **Supplementary Figures**

**Supplementary Fig. S1 Distinct gut microbiome composition before and after Kasai surgery**

A–C: Bar plots of the Chao1, Shannon, and Simpson diversity indices in patients before (Group_S1) and after (GroupS2) Kasai surgery. Statistical differences were analyzed using the Mann-Whitney test.

D: Heatmap showing the relative abundance of the top 15 genera in each sample.

**Supplementary Fig. S2 Alpha and beta diversity analysis were not significantly different, while there was a different composition and less species richness in S2_B relative to S2_A**

A: Heatmap showing the relative abundance of the top 15 genera in each S2A and S2B sample.

B–D: Bar plots showing the Chao1 and Shannon diversity index, and observed species in S2A and S2B group patients. The significance of differences were analyzed by Mann-Whitney test.

E: Beta diversity PCoA plot of Binary Jaccard distances in S2A and S2B group patients. The significance of differences was analyzed using the ADONIS test.

**Supplementary Fig. S3 Additional materials for Figure 4**

A–C: qPCR analysis of *IL1B*, *IL8*, and *KRT19* expression in BA organoids from the control and infection groups. The significance of differences was analyzed by Mann-Whitney test.

D: Western blot analysis of TGF-β1 levels in BA organoid lysates. Three samples (BA1615, BA1617, BA1619) were analyzed.

E: Relative TGF-beta1 protein levels were normalized to those of GAPDH, and the differences.

F: qPCR analysis of *CASP3*, *CASP9*, *BAX* expression in BA organoids from the control and infection groups.
